# Enhanced Diagnostic Efficiency of Endometrial Carcinogenesis and Progression in Women with Abnormal Uterine Bleeding through Peripheral Blood Cytokine Testing: A Multicenter Retrospective Cohort Study

**DOI:** 10.7150/ijms.91506

**Published:** 2024-01-21

**Authors:** Jincheng Ma, Dabin Liu, Xiaodan Mao, Leyi Huang, Yuan Ren, Xiaozhen Xu, Xiaoli Huang, Caiping Deng, Feifeng Shi, Pengming Sun

**Affiliations:** 1 Laboratory of Gynecologic Oncology, Fujian Maternity and Child Health Hospital, College of Clinical Medicine for Obstetrics & Gynecology and Pediatrics, Fujian Medical University, Fuzhou 350001, Fujian, China.; 2 Fujian Key Laboratory of Women and Children's Critical Diseases Research, Fujian Maternity and Child Health Hospital, Fuzhou 350001, Fujian, China.; 3 Fujian Clinical Research Center for Gynecological Oncology, Fujian Maternity and Child Health Hospital, Fuzhou 350001, Fujian, China.; 4 Department of Gynecology, Fujian Maternity and Child Health Hospital, College of Clinical Medicine for Obstetrics & Gynecology and Pediatrics, Fujian Medical University, Fuzhou 350001, Fujian, China.; 5 Zhangzhou Affiliated Hospital of Fujian Medical University, Zhangzhou 363000, Fujian, China.; 6 Department of Obstetrics and Gynecology, The First Affiliated Hospital, Fujian Medical University, Fuzhou 350005, Fujian, China.; 7 Department of Obstetrics and Gynecology, National Regional Medical Center, Binhai Campus of the First Affiliated Hospital, Fujian Medical University, Fuzhou 350212, Fujian, China.; 8 The Second Hospital of Nanping City, Nanping 354200, Fujian, China.

**Keywords:** endometrial cancer, cytokine, abnormal uterine bleeding, biomarker, inflammation, predictive model.

## Abstract

**Objective:** This study aimed to evaluate the role of plasma cytokine detection in endometrial cancer screening and tumor progression assessment in patients with abnormal uterine bleeding.

**Methods:** In this multicenter retrospective cohort study of 287 patients with abnormal uterine bleeding, comprehensive clinical information and laboratory assessments, including cytokines, routine blood tests, and tumor markers, were performed. Associations between the clinical indicators and endometrial carcinogenesis/progression were evaluated. The independent risk factors for endometrial cancer and endometrial cancer with deep myometrial invasion were analyzed using multivariate binary logistic regression. Additionally, a diagnostic model was used to evaluate the predictive efficacy of these identified risk factors.

**Results:** In patients with abnormal uterine bleeding, low IL-4 and high IL-8 levels were independent risk factors for endometrial cancer (p < 0.05). Combining IL-4, IL-8, CA125, and menopausal status improved the accuracy of assessing endometrial cancer risk. The area under curve of the model is 0.816. High IL-6 and IL-8 levels were independent risk factors for deep myometrial invasion in patients with endometrial cancer (p < 0.05). Similarly, combining IL-6, IL-8, and Monocyte counts enhanced the accuracy of assessing endometrial cancer risk with deep myometrial invasion. The area under curve of the model is 0.753.

**Conclusions:** Cytokines such as IL-4, IL-8, and IL-6 can serve as markers for monitoring endometrial cancer and its progression in women with abnormal uterine bleeding.

## Introduction

Endometrial cancer (EC) is the most prevalent gynecological malignancy globally 1. In 2020, China witnessed 81,964 newly reported EC cases, resulting in 16,607 fatalities 2, 3. Globally, the annual incidence of EC has been increasing year by year 4,5. Over 90% of EC patients manifest varying forms of abnormal vaginal bleeding as a primary symptom, encompassing postmenopausal vaginal bleeding and menstrual irregularities 6. Abnormal uterine bleeding (AUB), a prevalent symptom in obstetrics and gynecology 7. Among women aged 45 and older, those with AUB have a certain risk of EC 8. Distinguishing AUB from EC is of paramount importance for facilitating appropriate patient management.

Although procedures such as hysteroscopy, curettage, and endometrial biopsy exhibit high diagnostic accuracy, their invasive nature and associated discomfort, including intense pain, pose challenges 9, 10. The quest for a novel, non-invasive, and cost-effective diagnostic tool is imperative to mitigate unnecessary curettage. Peripheral blood samples, characterized by accessibility and relative noninvasiveness, have emerged as promising repositories for potential biomarkers. Although existing serum tumor markers such as HE4 and CA125 play a role in EC diagnosis, their sensitivity and specificity remain unclear 11, 12, 13. Cytokines, pivotal modulators of immune responses, offer heightened sensitivity compared to other immune factors. Previous studies have shown that increased serum levels of pro-inflammatory cytokines are associated with tumor metastasis and progression 14, 15.

However, screening methods employing immune blood biomarkers for prognosticating EC risk in patients with AUB are lacking. In response to this gap, our study propounds a pioneering approach that integrates blood cytokine testing to assess the propensity of EC incidence and progression in perimenopausal patients with AUB. This initiative aims to increase diagnostic precision and alleviate the clinical discomfort associated with diagnosis and treatment. Further, this study provides innovative avenues for advancing anti-inflammatory strategies and immunotherapeutic approaches in tumor management.

## Materials and Methods

### Ethics statement

Ethical approval was obtained from the Ethics Committee of Fujian Provincial Maternity and Children's Hospital (2022KYLLR03022). The requirement for informed consent was waived due to the retrospective nature of the study. The study protocol conformed with the principles of the Declaration of Helsinki.

### Study population

This retrospective cohort study was conducted at multicenter and included 287 patients aged 45 and older who presented to hospitals with AUB and underwent peripheral blood immune indicator testing from 2020 to 2022 in Fujian Province. Exclusion criteria included a history of inflammatory disease, autoimmune disease, chronic metabolic disease, chronic infectious disease, prior history of immunotherapy and other malignancies, and loss to follow-up. All patients underwent endometrial biopsy or curettage. Among them, 142 were diagnosed with EC. Within this group, 47 were associated with deep myometrial invasion (DMI), whereas 95 were not. The remaining 145 patients were diagnosed with benign uterine diseases.

### Clinical laboratory data

All patients underwent comprehensive clinical laboratory examinations, including assessments of cytokines, routine blood tests, and tumor markers. Data on risk factors for EC such as age, body mass index (BMI), and menopausal status were systematically collected. Additionally, histopathological findings were included in the study.

### Statistical analysis

Data analysis was performed using SPSS version 20.0, and R version 4.2.1. Continuous data were expressed as mean ± standard deviation (SD) or median and interquartile range (IQR). Student's t-test or Mann-Whitney U test was used to analyze the differences between groups. The chi-squared test or Fisher's exact test was used for categorical data, which were presented as counts with frequencies. Receiver Operating Characteristic (ROC) curve analysis was performed, and the ROC area under the curve (AUCs) was used to evaluate the diagnostic accuracy of EC. Logistic regression analysis was employed to identify independent risk factors and to establish and evaluate a diagnostic model. The significance level was set at p < 0.05.

## Results

### General characteristics between EC and Non-EC groups

**Figure 1:**
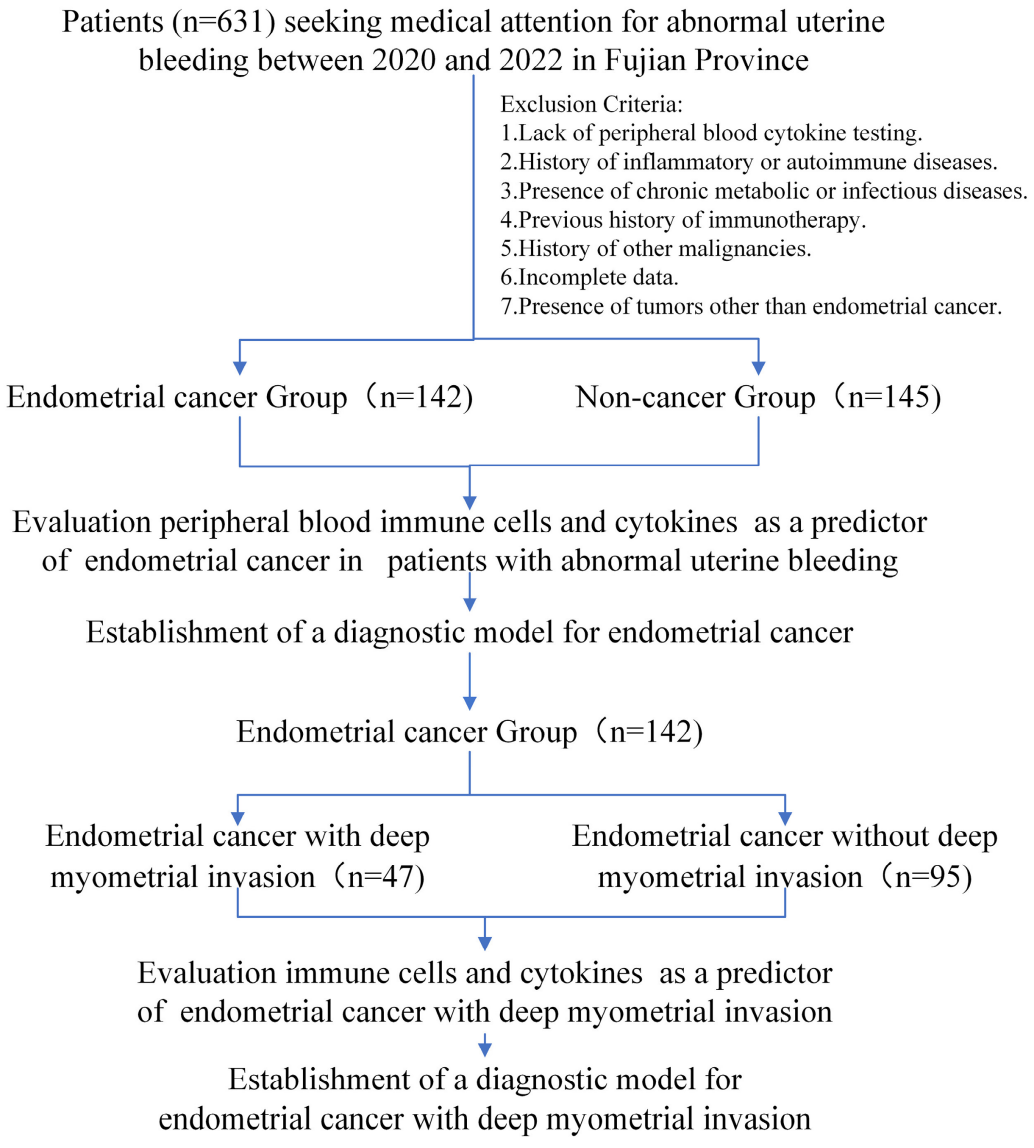
Study cohort flowchart.

A flowchart of the study is depicted in** Figure 1**. The outcomes unveiled a distinct pattern among perimenopausal individuals seeking medical care for AUB. Those diagnosed with EC tended to be of advanced age, predominantly in the postmenopausal phase (**Table 1**). Notably, the blood tumor marker CA125 was elevated in patients with EC (p < 0.05). This observation suggests a role for CA125 levels in evaluating the risk of EC in individuals presenting with AUB.

### Independent risk factors for endometrial carcinogenesis in AUB patients

**Figure 2:**
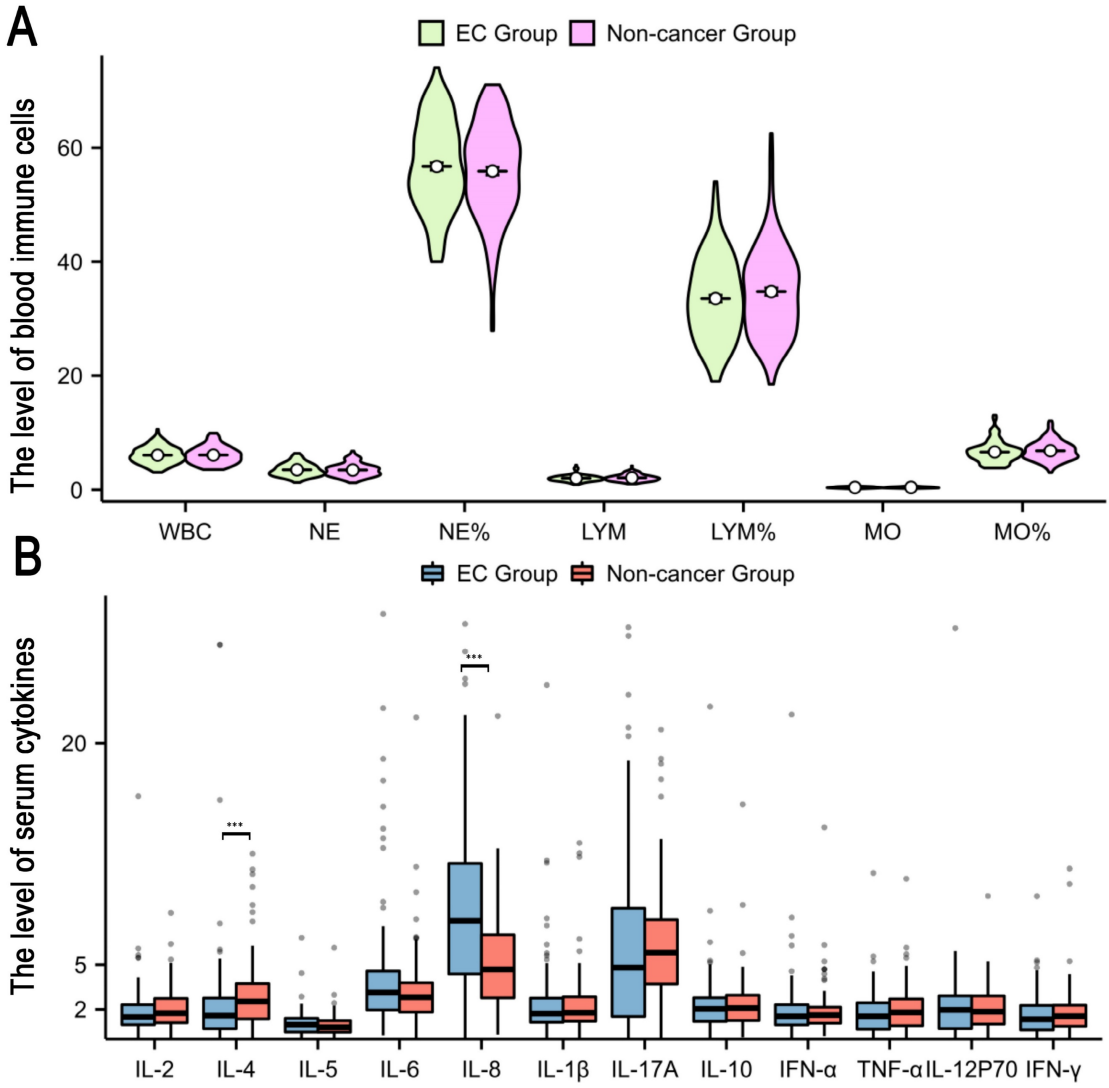
**Characteristics of peripheral blood immune cells and cytokines among study participants, comparing the endometrial cancer and Non-cancer group.** A: The peripheral blood immune cells between endometrial cancer and Non-cancer group; B: The peripheral blood cytokines between endometrial cancer and Non-cancer group. Note: *, p < 0.05; ***, p < 0.001; p<0.05 indicates significant differences. Abbreviations: WBC, white blood cell; NE, neutrophils; Mo, monocyte; Lym, lymphocyte; IL, Interleukin.

**Figure 3:**
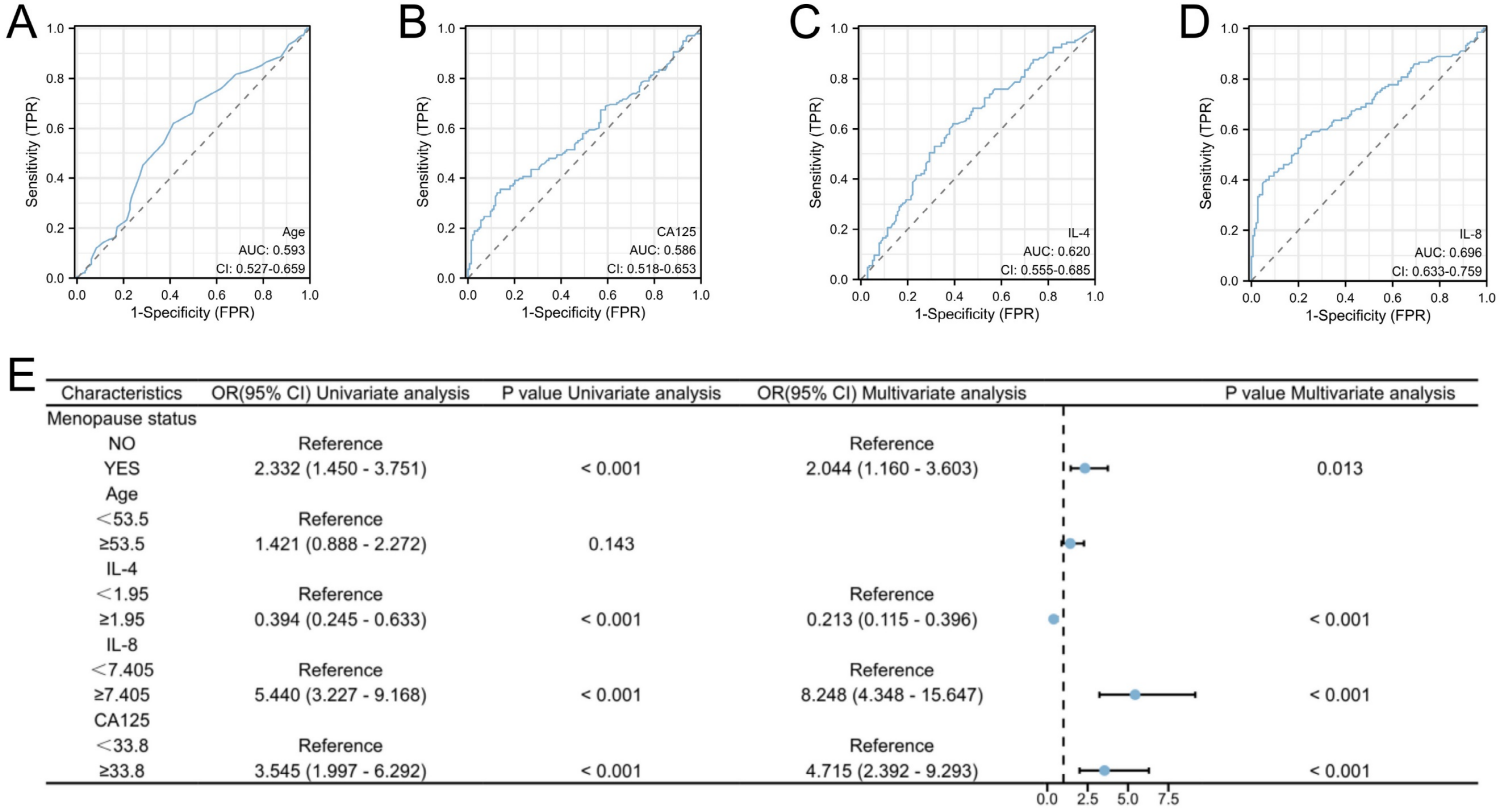
** High risk factors and predictive models for endometrial cancer in patients with abnormal uterine bleeding during perimenopause.** A-D: Receiver operating characteristic (ROC) curves for Age, IL-4, IL-8, and CA125. The cut-off values for these indicators were also determined (Age: Cutoff=53.5, IL-4: Cutoff=1.95, IL-8: Cutoff=7.405, and CA125: Cutoff=33.8); E: Logistic regression analyses for EC.

**Figure 4:**
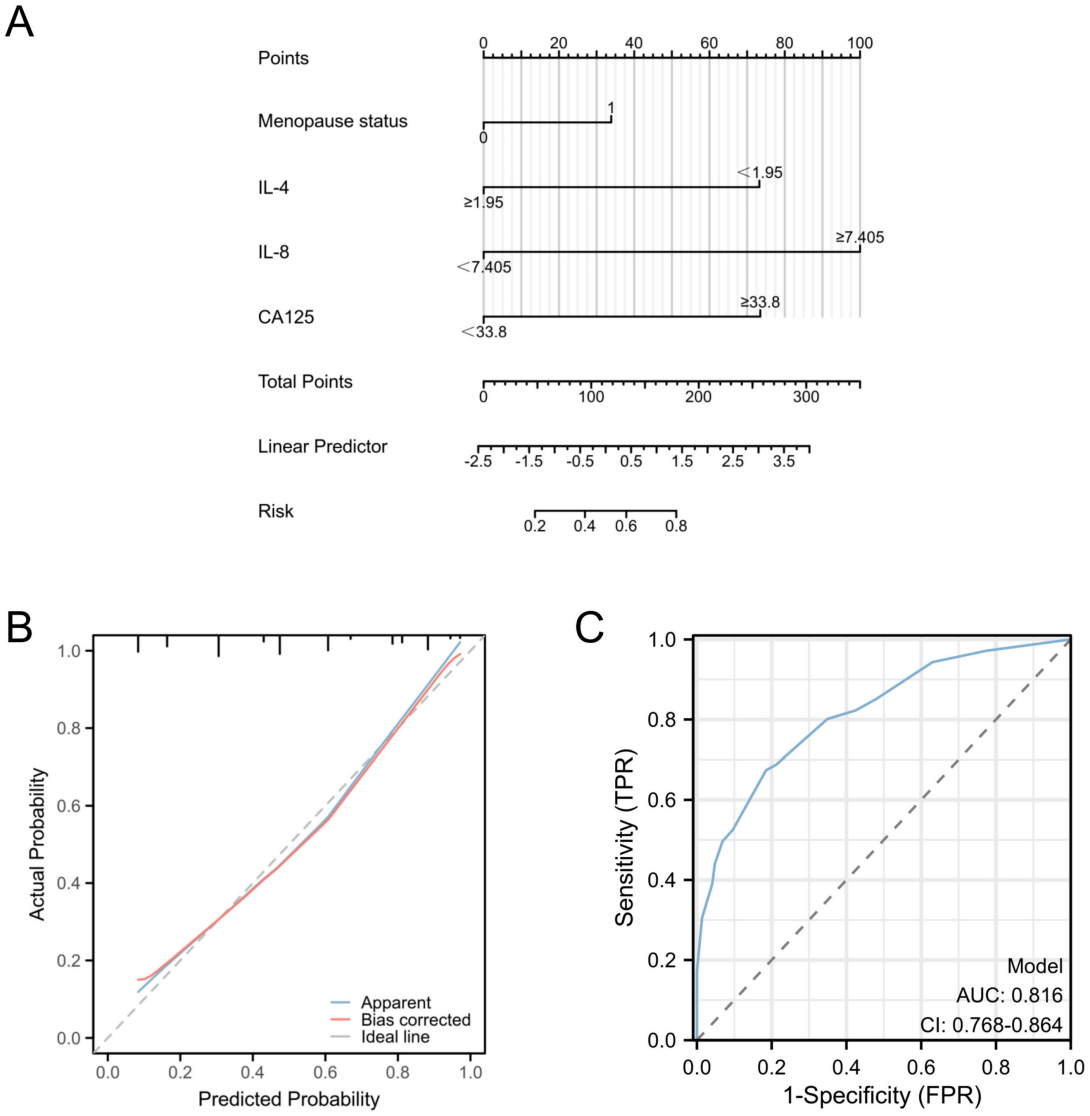
** Predictive models for endometrial cancer in patients with abnormal uterine bleeding during perimenopause.** A: Nomogram for predicting endometrial cancer; B: Calibration plots for the nomogram with Hosmer-Lemeshow P=0.8585; C: ROC curve for the nomogram. (AUC=0.816, p < 0.001).

Our investigation included the assimilation of routine blood test data and cytokine profiles from a patient cohort. The assessment of neutrophils, lymphocytes, and monocytes levels revealed no statistically significant differences between the EC and non-cancerous groups. This observation implies a limited role for immune cells in routine blood assessments of tumors. Conversely, in the EC group, the pro-inflammatory marker IL-8 exhibited a notable increase (p < 0.05), whereas the anti-inflammatory cytokine IL-4 displayed a distinct decrease (p<0.05), and these disparities were statistically significant (**Figure 2**). The statistically significant indicators were subsequently evaluated by dichotomizing continuous data using ROC curve analysis and the Youden Index (**Figure 3A-D**). Cutoff values were established for significant EC group indicators, including age (cutoff = 53.5), IL-4 (cutoff =1.95), IL-8 (cutoff =7.405), and CA125 (cutoff =33.8). Comprehensive univariate and multivariate logistic regression analyses were then performed to determine baseline characteristics and clinicopathological features. The resultant outcomes revealed that menopausal status and IL-4, IL-8, and CA125 levels were independent risk factors for the EC-group (all p < 0.05, **Figure 3E**). These independent risk factors were harnessed to construct a nomogram tailored for the EC group, thus offering a quantitative prediction avenue (**Figure 4A**). Calibration plots corroborated a commendable alignment between actual observations and predicted probabilities, substantiated by a Hosmer-Lemeshow P-value of 0.8585 (**Figure 4B**). Concurrently, the ROC curve analysis indicated that, when compared to individual indices, the amalgamation of menopausal status, IL-4, IL-8, and CA125 indices significantly augmented the diagnostic efficacy for EC patients (AUC=0.816, p < 0.001, **Figure 4C**).

### General characteristics between EC with and without deep myometrial invasion

The primary mode of metastasis in EC involves direct spread, with cancer cells infiltrating the myometrium, leading to further expansion, invasion, and subsequent metastasis. Consequently, the categorization of patients with EC into those with and without deep myometrial invasion serves as a means of investigating risk factors for tumor progression. Basic data pertaining to EC cases with and without deep myometrial invasion are detailed in **Table 2**. Notably, a statistically significant difference were observed between the two groups in terms of Federation International of Gynecology and Obstetrics (FIGO) stage, grading, and lymph node metastasis. These indicators directly correlated with deep myometrial invasion and tumor progression, thus underscoring their limited research value. Conversely, CA125 levels were not significantly different between the two groups. This observation suggests a restricted role of CA125 in determining the trajectory of tumor progression.

### Independent risk factors for EC with deep myometrial invasion and without patients with AUB

**Figure 5:**
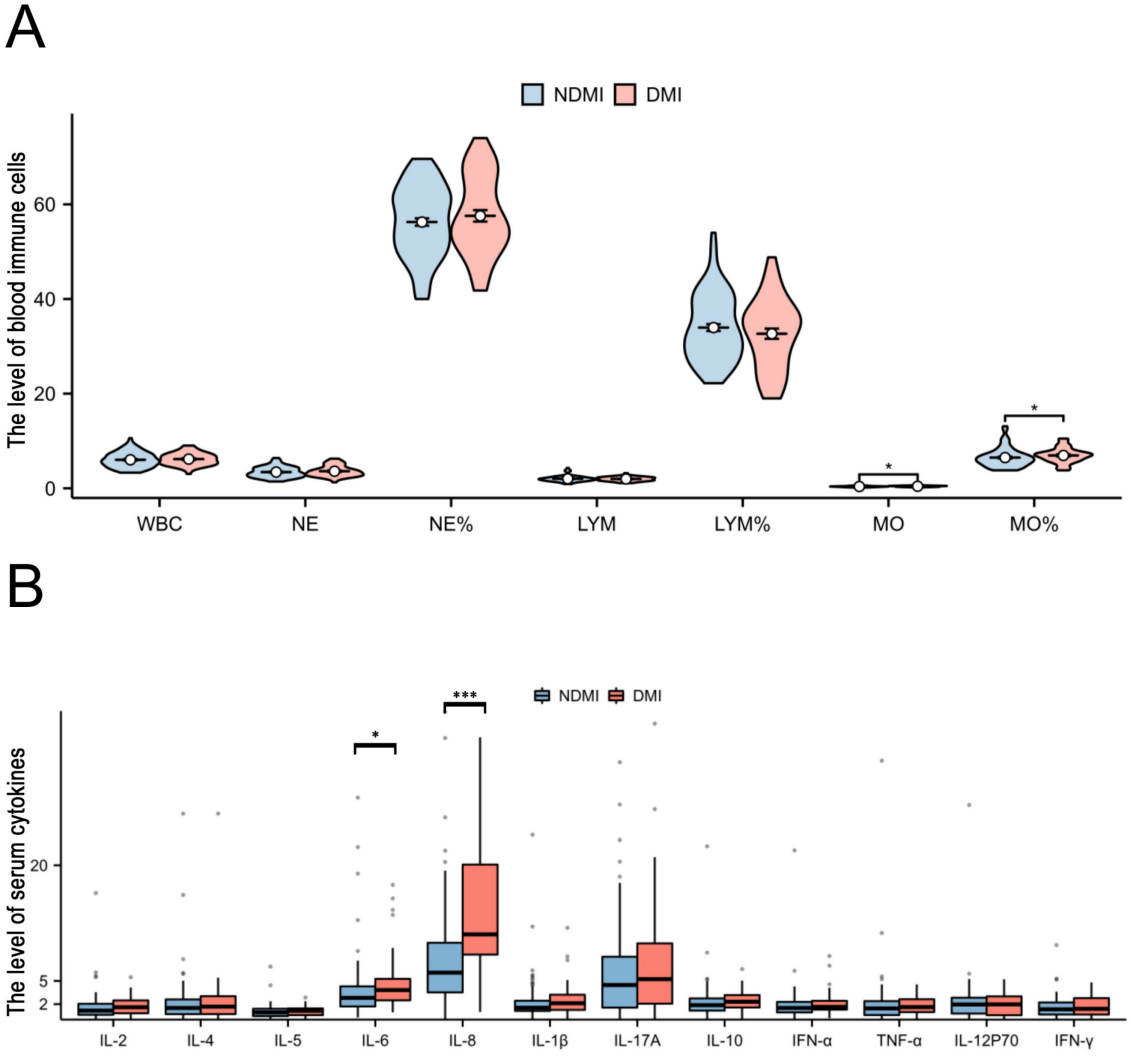
** Characteristics of peripheral blood immune cells and cytokines between endometrial cancer with deep myometrial invasion and without deep myometrial invasion in study participants.** A: The peripheral blood immune cells between endometrial cancer with and without deep myometrial invasion; B: The peripheral blood cytokines between endometrial cancer with and without deep myometrial invasion. Note: *, p < 0.05; ***, p < 0.05; p<0.05 indicates significant differences. Abbreviations: WBC, white blood cell; NE, neutrophils; Mo, monocyte; Lym, lymphocyte; IL, interleukin.

**Figure 6:**
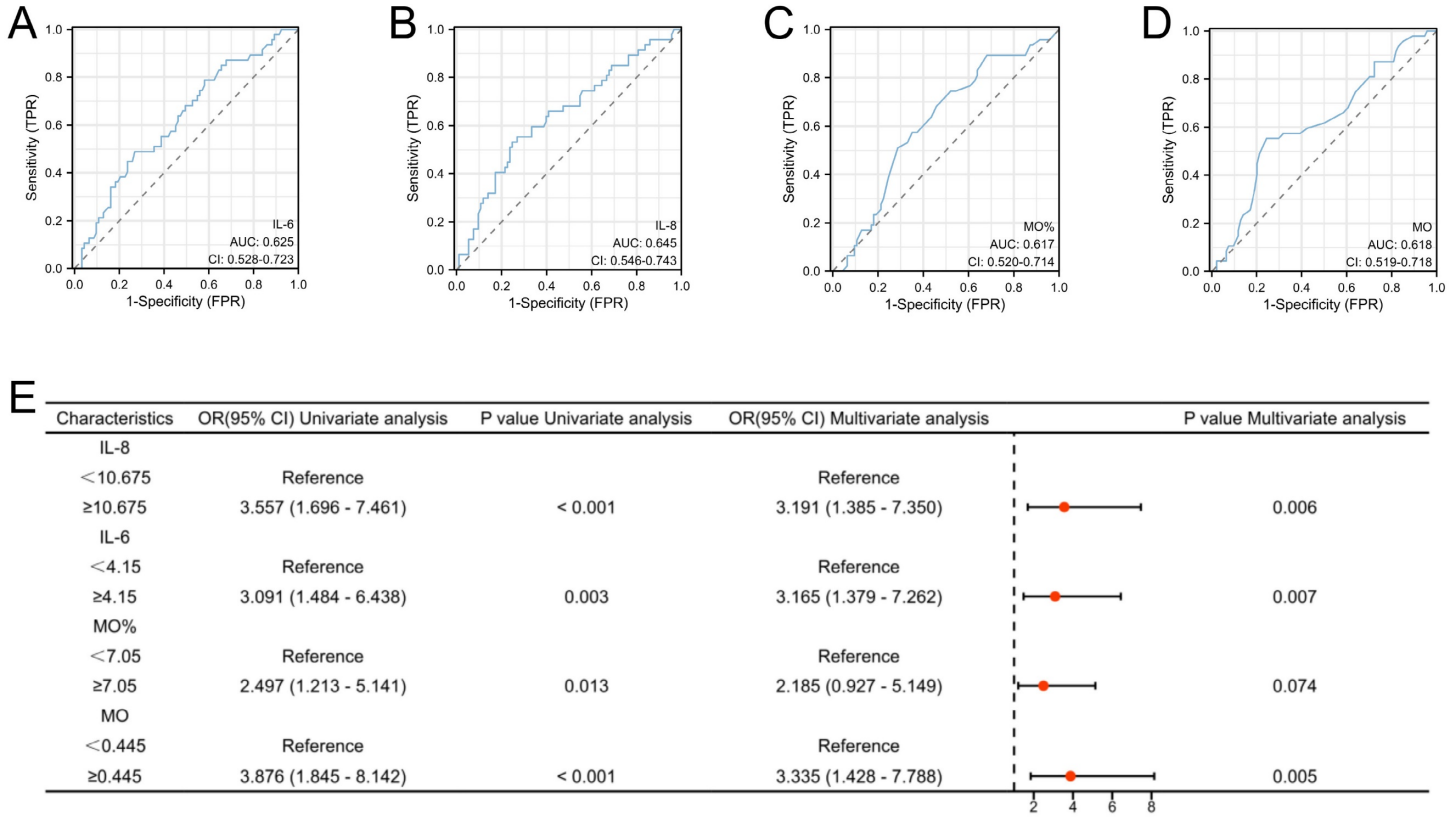
** High risk factors for endometrial cancer with deep myometrial invasion in patients with abnormal uterine bleeding.** A-D: Receiver operating characteristic (ROC) curves for IL-6, IL-8, Mo, and Mo. The cut-off values for these indicators were also determined (MO: Cutoff=0.445), MO%: Cutoff=7. 05, IL-6: Cutoff=4.15, and IL-8: Cutoff=10.675). E: Logistic regression analyses for endometrial cancer with deep myometrial invasion.

Compared to cases of EC devoid of deep myometrial invasion, both monocyte count and percentage were considerably elevated in EC cases characterized by deep myometrial invasion. Additionally, within the subset of EC cases marked by deep myometrial invasion, elevated levels of the pro-inflammatory markers IL-6 and IL-8 were apparent. All the observed differences were statistically significant (**Figure 5**). The highlighted significant parameters were subsequently subjected to further analysis. Continuous data were dichotomized for subsequent analyses using ROC curve analysis and the Youden Index (**Figure 6A-D**). Consequently, distinct cutoff values were established for monocyte count (MO) (cutoff =0.445), MO % (cutoff =7.05), IL-6 (cutoff =4.15), and IL-8 (cutoff =10.675).

**Figure 7:**
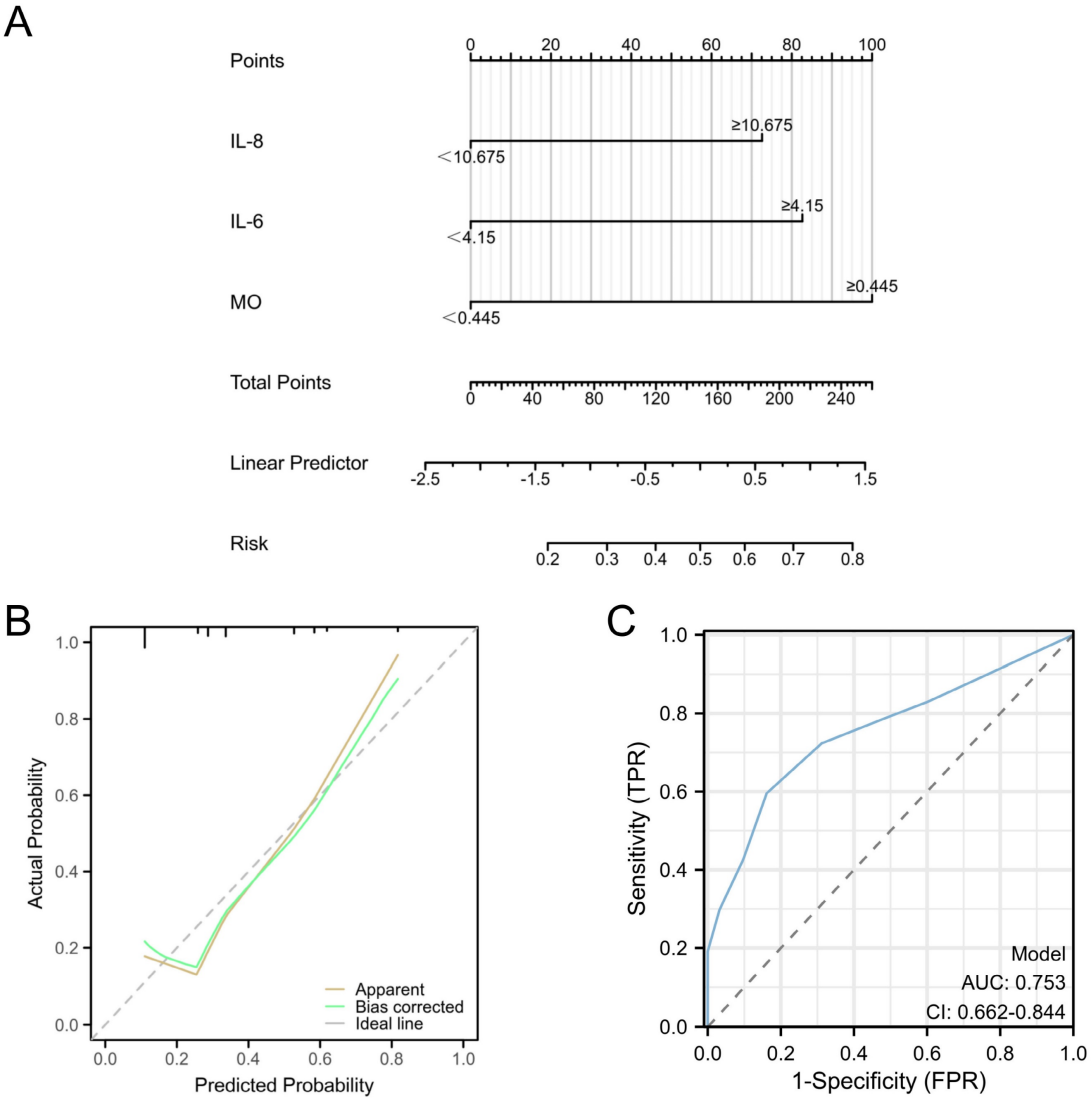
** Predictive models for EC with deep myometrial invasion in EC patients.** A: Nomogram for predicting endometrial cancer with deep myometrial invasion. B: Calibration plots for the nomogram, Hosmer-Lemeshow P=0.8102. C: ROC curve for the nomogram, (AUC=0.753, p < 0.001).

Univariate and multivariate logistic regression analyses were performed for all baseline and clinicopathological features. The outcomes underscored the independence of MO, IL-6, and IL-8 as risk factors for EC with deep myometrial invasion (all p < 0.05; **Figure 6E**). These significant indicators were used to construct a nomogram tailored to predict EC cases featuring deep myometrial invasion, offering a quantitative prediction avenue (**Figure 7A**). Notably, calibration plots affirmatively demonstrated robust consistency between actual observations and predicted probabilities, as evidenced by a Hosmer-Lemeshow P-value of 0.8102 (**Figure 7B**). Concurrently, ROC curve analysis highlighted the potency of combining MO, IL-6, and IL-8 over individual indices, significantly augmenting the diagnostic efficacy for EC cases with deep myometrial invasion (AUC=0.753, p < 0.001, **Figure 7C**).

## Discussion

AUB is a prevalent symptom that demands attention in women's health and is frequently associated with the potential risk of EC. Regrettably, existing screening approaches are either invasive or lack the requisite specificity 16, 17. Such diagnostic uncertainties inflict both physical and psychological distress on patients with AUB. A significant challenge for clinicians is distinguishing between benign AUB and EC through non-invasive methodologies.

In our study, the analysis of cytokine test data, particularly IL-8, IL-4, and IL-6 levels, proved noteworthy in the diagnosis of EC among individuals with AUB. In patients with AUB, low IL-4 and high IL-8 levels were identified as independent risk factors for EC. Combining IL-4, IL-8, CA125, and menopausal status enhanced the accuracy of EC risk assessment. Furthermore, high IL-6 and IL-8 levels were independent risk factors for deep myometrial invasion in patients with EC. The combination of IL-6, IL-8, and monocyte counts could provide a more accurate assessment of the risk of EC with deep myometrial invasion.

The role of inflammation in tumorigenesis and tumor development has received extensive attention, as it influences tumor initiation, growth, progression, and metastasis 18. IL-6 and IL-8 are known pro-inflammatory cytokines, whereas IL-4 is an anti-inflammatory cytokine 19. Our research indicates that the pro-inflammatory factor IL-8 was elevated in patients with EC. Studies have demonstrated increased levels of IL-8 in various tumor tissues and patient serum, including breast cancer, nasopharyngeal carcinoma, colorectal cancer, and gastric cancer 20. Numerous previous studies have demonstrated increased levels of IL-8 in patients with EC when compared in the normal population 21, 22, 23. Kotowicz et al. suggested that high levels of IL-8 in the serum can serve as an indicator for distinguishing patients with EC from the normal population, with a diagnostic sensitivity of 0.818 21.

The high expression of IL-8 is not only related to the occurrence of EC but also to its progression and prognosis. Previous studies have shown a statistically significant correlation between EC myometrial invasion and IL-8 levels 23, 24. IL-8 appears to function as an angiogenic switch during myometrial invasion in stage I uterine ECs 23, 24. Thus, it may serve as a valuable marker for stratifying patients with EC based on lymphovascular invasion and EC grade 23. Similarly, our research indicate that IL-8 was an independent risk factor for deep myometrial invasion in patients with EC. Kotowicz et al. 21 demonstrated the clinical utility of IL-8 measurements as potential prognostic factors in type 1 EC. In that study, elevated pretreatment IL-8 serum levels were independently associated with shorter disease-free and overall survival. Furthermore, a direct link between high serum IL-8 concentrations and disease progression, such as tumor size, stage, and prognosis, has been reported in patients with breast, colon, ovarian, prostate, and melanoma 25, 26. These associations may be attributed to IL-8's role in promoting angiogenesis, cancer stem cell survival signaling, and immunosuppression 27.

In our study, IL-6, another pro-inflammatory cytokine, exhibited increased expression in the EC deep myometrial invasion group. Serum IL-6 levels were an independent risk factor for poor prognosis. This result is consistent with other findings that IL-6 might significantly predict cancer progression in colorectal, prostate, and breast cancer 28. High serum IL-6 levels might promote the progression of EC, possibly related to IL-6-induced migration and invasion of EC cells via the Stat3 signaling pathway 29. Additionally, previous studies have shown that IL-6 promotes EC growth through an autocrine feedback loop involving ERK-NF-κB signaling pathway 30. In addition to promoting the EC cell growth, IL-6 is upregulated in EGFR-mutant non-small cell lung cancer, where it suppresses T- and NK-cell functions. IL-6 blockade enhances antitumor immunity and the efficacy of anti-PD-1 therapy 31.

IL-4, a potent regulator of antitumor immune responses, possesses both tumor-promoting and tumor-inhibiting properties due to its immunosuppressive and anti-angiogenic functions 32, 33. In our study, we observed a decrease in IL-4 levels in patients with EC. Consistent with our findings, IL-4 is also identified to exert an inhibitory influence on the proliferation of renal, colon, and breast cancer cells. This influence further elicits regression in specific tumor xenografts within murine cancer models 34. Similarly, certain studies have shown that IL-4 has anti-inflammatory effects and may decrease the risk of esophageal squamous cell carcinoma by inhibiting inflammation 35, 36. In contrast, some studies have shown that higher serum IL-4 levels correlate with advanced cancers 37, 38. However, studies on the role of IL-4 in EC are limited. Additionally, polymorphisms of the IL-4 gene also determine its complex role in tumors, necessitating more detailed research.

The reduction of the anti-inflammatory cytokine IL-4 and the elevation of the pro-inflammatory factors L-6 and IL-8 in EC contributed to understand the intricate relationship between inflammation and cancer. Therefore, these changes could serve as monitoring indices for endometrial carcinogenesis and progression in women with AUB. Numerous studies have shown the pivotal role of other cytokines in the diagnosis and treatment of EC. Anti-inflammatory cytokines, including IL-10, TGF-β, IL-1Ra, and IDO, facilitate the evasion of immune attacks by the tumor. They establish an immunomodulatory environment, effectively suppressing the immune system. Pro-inflammatory cytokines such as IL-6, IL-8, IL-31, and IL-33 promote tumor growth and resistance to apoptosis 39. Dinkic et al. demonstrated the potential involvement of cytokine IL-6 along with chemokines CCL5 and CCL2 in contributing to chemoresistance in EC 40. Thus, cytokines have emerged as important targets for the diagnosis and treatment of EC.

Monocytes play an important role in various inflammatory responses in the body, including acute and chronic inflammation, tumors, and immune inflammation. Monocytes participate in the transmission of inflammatory signals and the generation and secretion of inflammatory molecules, thereby affecting the development of inflammatory reactions 41. In our study, monocyte counts were considerably elevated in EC cases characterized by deep myometrial invasion. Matsuo et al. documented a significant correlation linking elevated monocyte counts to heightened risks of deep myometrial tumor invasion, pelvic lymph node metastasis, advanced-stage disease, and reduced survival rates in patients diagnosed with EC 42. Analogous correlations between elevated circulating monocyte counts and diminished survival have been noted in additional gynecological malignancies, encompassing ovarian and cervical cancers 43. Moreover, patients diagnosed with EC have displayed altered functionality in circulating monocytes, notably exhibiting heightened expression of the vascular endothelial growth factor (VEGF) receptor 1 on their cell surface. VEGF, produced by the tumor, exerts paracrine effects on macrophages, shaping them into tumor-associated macrophages (TAMs) in the local tumor microenvironment. Additionally, VEGF potentially have distant effects that modulate global monocyte functions in EC 44.

Numerous cancers either originate from chronic inflammation or prompt an inflammatory reaction (termed tumor-elicited inflammation), thereby establishing inflammation as a facilitating hallmark of cancer 18, 45. Inflammation contributes to carcinogenesis by directly inducing mutagenesis or stimulating cytokine responses 18. Further, cytokines recruit monocytes to become TAMs, with monocytic macrophages being the main immune cells producing cytokines. This, in turn, amplifies the role of inflammation.

Cumulatively, these findings accentuate the inflammatory backdrop associated with patients susceptible to EC, where inflammation potentially serving as a catalyst for cancer development. Cytokines play a vital role in mediating inflammation and cancer development. Therefore, cytokines hold promise as potential biomarkers for the early screening and continuous monitoring of tumor progression in EC. The identification of peripheral blood cytokines yields valuable insights into the intricate interplay between inflammation, immunity, and tumorigenesis. This provides innovative avenues for advancing anti-inflammatory strategies and immunotherapy approaches in tumor management.

Acknowledging the limitations of this study is crucial. The inclusion of a limited sample size, compounded by the nascent adoption of cytokine testing in clinical practice, may have introduced bias. Consequently, cautious interpretation is necessary, underscoring the need for further exploration using expanded cohorts to validate our conclusions. Moreover, the retrospective design of our study mandates vigilance regarding potential biases and limitations in data collection and interpretation. A prospective approach could provide more robust evidence. In future studies, these limitations could be circumvented through the inclusion of a broader and more diverse patient cohort, accompanied by comprehensive data collection to facilitate external validation.

## Conclusions

Cytokines such as IL-4, IL-8, and IL-6 can be used to monitor endometrial carcinogenesis and progression in women with AUB.

## Figures and Tables

**Table 1:** Baseline characteristics of EC Group and Non-cancer Group in study participants.

Characteristics		EC Group	Non-cancer Group	P value
N		142	145	
**Age, median (IQR)**		55 (51, 61)	52 (48, 58)	**0.006**
**BMI, median (IQR)**		24.321 (22.022, 27.441)	23.712 (21.644, 26.839)	0.379
**Menopause status, n (%)**	**YES**	92 (32.1%)	64 (22.3%)	**<0.001**
	**NO**	50 (17.4%)	81 (28.2%)	
**History of hypertension, n (%)**	**YES**	46 (16%)	39 (13.6%)	0.289
	**NO**	96 (33.4%)	106 (36.9%)	
**History of diabetes, n (%)**	**YES**	26 (9.1%)	25 (8.7%)	0.791
	**NO**	116 (40.4%)	120 (41.8%)	
**CA125, median (IQR)**		20 (11.975, 41.425)	17.2 (11.55, 27.25)	**0.021**

**Note:** p < 0.05 was considered to be statistically significant. **Abbreviations: BMI**, body mass index; **CA125**, cancer antigen 125.

**Table 2:** Baseline characteristics of endometrial cancer group participants stratified by deep muscle invasion and non-deep muscle invasion.

Characteristics		DMI	NDMI	P value
N		47	95	
Age, mean ± sd		57.213 ± 9.5734	55.211 ± 7.8482	0.186
BMI, median (IQR)		24.524 (22.49, 27.319)	24.051 (21.967, 27.584)	0.862
Menopause status, n (%)	Yes	30 (21.3%)	61 (43.3%)	0.901
	No	17 (12.1%)	33 (23.4%)	
History of hypertension, n (%)	Yes	16 (11.3%)	30 (21.3%)	0.799
	NO	31 (22%)	64 (45.4%)	
History of diabetes, n (%)	Yes	7 (5%)	19 (13.5%)	0.443
	NO	40 (28.4%)	75 (53.2%)	
FIGO stage, n (%)	Ⅰ~Ⅱ	36 (25.4%)	95 (66.9%)	< 0.001
	Ⅲ~Ⅳ	11 (7.7%)	0 (0%)	
Grade, n (%)	G1 G2 G3	22 (15.5%) 19 (13.4%) 6 (4.2%)	76 (53.5%) 13 (9.2%) 6 (4.2%)	< 0.001
Deep muscle invasion, n (%)	Yes	47 (33.1%)	0 (0%)	< 0.001
	No	0 (0%)	95 (66.9%)	
Lymph node metastasis, n (%)	No	34 (23.9%)	95 (66.9%)	< 0.001
	Yes	13 (9.2%)	(0%)	
ER, n (%)	- ~+	33 (23.2%)	54 (38%)	0.124
	++~+++	14 (9.9%)	41 (28.9%)	
PR, n (%)	- ~+	35 (24.6%)	56 (39.4%)	0.070
	++~+++	12 (8.5%)	39 (27.5%)	
Ki67, median (IQR)		0.4 (0.3, 0.6)	0.4 (0.3, 0.4)	0.106
CA125, median (IQR)		21.6 (15.24, 42.9)	18.27 (11.1, 40.325)	0.232

**Note:** p<0.05 was considered to be statistically significant. **Abbreviations: DMI,** deep muscle invasion**; BMI**, body mass index; **FIGO**, International Federation of Gynecology and Obstetrics;** ER**, estrogen receptor; **PR**, progesterone receptor; **CA125**, cancer antigen 125.

## Abbreviations

AUB: abnormal uterine bleeding
EC: endometrial cancer
NK: natural killer
BMI: body mass index
SD: standard deviation
IQR: interquartile range
ROC: receiver operating characteristic
AUC: areas under the curve
BMI: body mass index
ORs: odds ratios
CIs: confidence intervals
WBC: white blood cell
NE: neutrophils
Mo: monocyte
Lym: lymphocyte
IL: interleukin
FIGO: international federation of gynecology and obstetrics
DMI: deep myometrial invasion
CA125: carbohydrate antigen 125
TCGA: The Cancer Genome Atlas
ER: estrogen receptor
PR: progesterone receptor
TAMs: tumor-associated macrophages
